# Opportunities and Gaps in Primary Care Preventative Health Services for Transgender Patients: A Systemic Review

**DOI:** 10.1089/trgh.2016.0019

**Published:** 2016-11-01

**Authors:** E. Kale Edmiston, Cameron A. Donald, Alice Rose Sattler, J. Klint Peebles, Jesse M. Ehrenfeld, Kristen Laurel Eckstrand

**Affiliations:** ^1^ Vanderbilt Program for LGBTI Health, Nashville, Tennessee.; ^2^ Columbia University Program in Narrative Medicine, New York, New York.; ^3^ University of California San Francisco School of Medicine, San Francisco, California.; ^4^ Department of Dermatology, University of Wisconsin School of Medicine and Public Health, Madison, Wisconsin.; ^5^ Vanderbilt University Medical Center, Nashville, Tennessee.; ^6^ Department of Psychiatry, University of Pittsburgh, Pittsburgh, Pennsylvania.

**Keywords:** HIV, primary care, sexual health, transgender

## Abstract

**Purpose:** Transgender people face barriers to accessing healthcare, resulting in population-level disparities in health outcomes. Little research is available to better understand the receipt of primary healthcare among transgender patients or how the rate of receipt of preventive care may differ among transgender populations.

**Methods:** The medical literature regarding U.S. adult transgender primary healthcare was reviewed using a keyword search strategy: transgender OR transsexual OR transvestite OR gender nonconforming for articles published between January 1, 2001 and June 15, 2015. Studies addressing the following topics as assessed by the Behavioral Risk Factor Surveillance System were extracted for qualitative review: colorectal cancer screenings, mammography or chest/breast tissue examinations, cholesterol and blood pressure screenings, tobacco use and smoking cessation, cervical cancer or human papillomavirus (HPV) screenings, human immunodeficiency virus (HIV), annual flu shot, and insurance coverage.

**Results:** The search identified 1304 eligible records, of which 41 discussed transgender primary or preventive care. The majority of studies discussed HIV rates or risk behaviors, while fewer articles addressed pelvic examinations, tobacco use, insurance coverage, and cholesterol screenings. No studies addressed mammography or chest/breast tissue examinations, colorectal screenings, or flu shots.

**Conclusions:** Findings from articles addressing five topics are discussed: HIV, cholesterol screenings, tobacco use, pelvic health, and insurance coverage. Gaps in the extant literature, including the lack of studies of nonbinary people, transgender men of color, and transgender people living outside of large coastal urban centers, are discussed. This review, coincident with other health disparity findings, suggests an urgent need for research that addresses the primary care needs of all transgender and gender nonconforming people.

## Introduction

There are significant disparities in access to and receipt of healthcare among transgender and gender nonconforming populations. Limited availability of appropriate and competent care and differential access to inclusive health insurance perpetuate these disparities and lead to significant morbidity and mortality.^1^ Although much research has focused on the transition-related care needs of transgender people, access to primary care is critical and is understudied.^2^ Primary care is the first point of entry into the healthcare system for most undiagnosed patients and is care that is not differentiated on the basis of problem origin or diagnosis. Primary care encompasses care for most basic medical needs and also includes health education, health maintenance, and disease prevention, in addition to diagnosis and treatment.^3^ Thus, access to primary care is a major determinant of health equity.^4^

Despite data suggesting widespread health disparities, human immunodeficiency virus (HIV) status is perhaps the best-characterized preventable health condition among transgender populations, with multiple studies showing an increased incidence of HIV and acquired immune deficiency syndrome (AIDS) among transgender populations, particularly transgender women of color.^5–7^ High rates of HIV/AIDS among transgender people are likely due to multiple interacting factors, including economic marginalization, social stigma, and limited access to healthcare and HIV screening.^8^ Transgender people are also generally less likely to access preventive health screenings.^9^ The reasons for reduced receipt of preventive health screenings are complex and may be due in part to social and economic marginalization, fear of discrimination, lack of access to providers experienced in treating transgender people, lack of provider awareness of appropriate screening guidelines for transgender people, as well as the absence of evidence-based screening guidelines from expert bodies and professional societies. Systemic barriers and discrimination also contribute to health disparities, as transgender people are more likely to experience violence, homelessness, and unemployment than cisgender people, leading to overall poorer health outcomes.^5,6,10–12^

Reduced access to comprehensive health insurance persists despite recent national efforts to reduce these barriers. Transgender people are more likely to be on public insurance than the general U.S. population, and transgender people on public insurance are more likely to be denied care due to their gender identity than those with private insurance.^9,13^ Among those who do have insurance, coverage is variable based on concordance of sex assigned at birth and gender identity, and type of medical care sought.^14^ Combined with the well-documented delay and avoidance of care due to fear of discrimination, these barriers amplify difficulties transgender individuals face when prioritizing their health.^9^

Although an increasing number of studies demonstrate health disparities among transgender communities, many of these studies were performed at single transgender health centers and/or using local convenience samples that limit the generalizability of the results. Furthermore, while recent reviews have discussed barriers to care for and experience of mental health, no reviews have focused on primary care needs for transgender populations.^15,16^ Reviews of HIV prevalence among transgender people have not included information regarding HIV rates among transgender men.^7,17–20^ Given the nationwide distribution of individuals identifying as transgender and the role of primary care in addressing preventive health, there is a need to better characterize current delivery of care to transgender individuals. In this systematic review, the medical literature regarding primary care among transgender people was reviewed to understand the diverse needs of transgender communities. Specifically, we were interested in key primary care measures as assessed by the Center for Disease Control's Behavioral Risk Factor Surveillance System (BRFSS) and rates of insurance coverage. We were also interested in determining if the literature reflected the diversity of transgender people with regard to race, gender identity, sexual orientation, and geographic location. We hypothesized that the literature would show significant disparities in rates of receipt of primary care, as well as high rates of risk behaviors such as unprotected sex and smoking. Secondarily, we hypothesized that transgender people from rural areas and the South of the United States would be underrepresented in the healthcare literature relative to transgender people from coastal urban areas.

## Methods

### Search strategy and selection criteria

We performed a systematic review for all peer-reviewed published studies containing information related to healthcare in transgender and gender nonconforming populations. We searched Pubmed and Web of Knowledge using a combined heading search strategy: “transgender” OR “transsexual” OR “transvestite” OR “gender non-conforming” between January 1, 2001 and June 15, 2015. The search was restricted to publications written in the English language. We reviewed reference lists of retrieved articles to identify other relevant studies for review. We did not include data from abstracts, opinions, reviews, or unpublished studies.

Multiple authors (E.K.E., A.R.S., J.K.P., K.L.E., C.A.D.) reviewed the retrieved studies to first confirm that the study did, in fact, discuss transgender populations. Studies were included for analysis if the study was performed in the United States, if the sample consisted of adults (18 years of age or older), and was observational in design. Case studies were not included in the analysis. Given the purpose of the review in examining primary care of transgender individuals, studies assessing any factors related to the following health measures as indicated by BRFSS were included: smoking status, HIV status, colorectal screening (age >50), mammography (age >50), breast examination (age >18), cervical human papillomavirus (HPV) screening (age >18), cholesterol screening (age >18), annual flu shot (age >18), or insurance status.^21^

For each study, we extracted the sample *n*, the location of the study, the percentage of the sample identified as people of color, and the gender identity makeup of the transgender sample, as well as the comparison sample if applicable (cisgender women or cisgender men). We also extracted the study methods (computer-based survey, structured, in-person interview, laboratory testing, including histopathological methods, or chart review) and the primary findings. For primary findings, we extracted and reported odds ratios and *p*-values when available, but, because many studies were observational in nature and thus did not include statistical analyses, we also report mean incidence or prevalence rates as well.

## Results

**FIG. 1.**
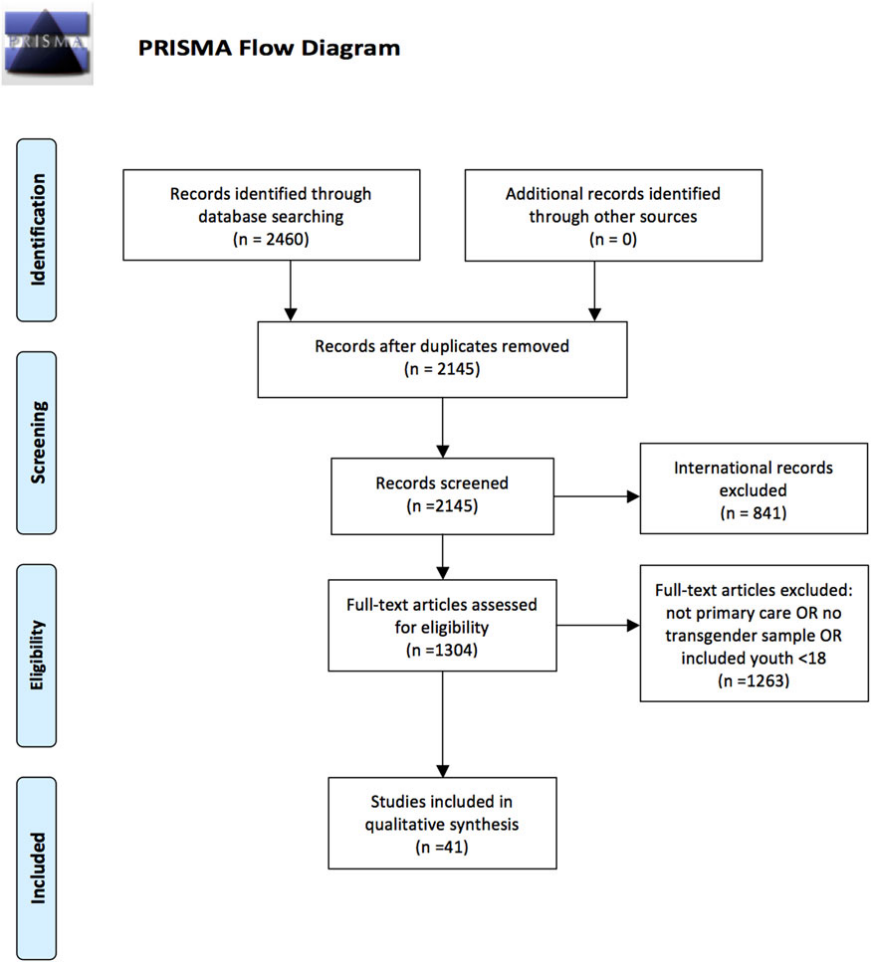
PRISMA flow diagram. From Moher D, Liberati A, Tetzlaff J, Altman DG, The PRISMA Group. *P*referred *R*eporting *I*tems for *S*ystematic *R*eviews and *M*eta-*A*nalyses: The PRISMA Statement. PLoS Med. 2009;6:e1000097. DOI:10.1371/journal.pmed1000097.

We identified 2460 studies meeting initial keyword search and eligibility criteria. Three hundred fifteen articles were duplicated in our compiled reference list. Of the remaining 2145 articles assessed, 841 were studies of international samples. One thousand three hundred four studies were assessed for eligibility; 455 did not include data on a distinct transgender sample either because they were (1) unrelated to the transgender community, but were instead studies of cisgender gay, lesbian, and/or bisexual (LGB) people, or (2) did not distinguish between cisgender gay men and transgender women in statistical analysis. An additional 802 did not address primary care. Of the remaining 47 studies, 6 included mixed samples of youth under the age of 18 and adults. Forty-one unique articles were included in our qualitative systematic analysis. The majority (*n*=31) pertained to HIV prevalence, eight discussed insurance status, eight discussed smoking, two were related to cholesterol screening, and four discussed pelvic examinations/Pap tests, nonexclusively. None of the included studies assessed flu vaccination, colorectal screening, or breast cancer screenings (Fig. 1).

### Sample characteristics

Overall, 2 of the 41 articles included samples that were not exclusively urban dwelling.^10,22^ Twenty-one articles included samples from the West Coast,^8,11,23–41^ 17 included samples from the East Coast,^12,22,29,38,42–54^ 3 from the Midwest,^8,29,38^ 3 from the South,^10,55,56^ and 1 each from Hawaii^57^ or Mountain states^29^ (multiple studies included samples from more than one region). One study included a national sample from an online survey and did not specify the location of participants.^58^

Most studies were conducted in samples of transgender women only (*n*=18),^8,11,12,23,30–33,36,37,40,41,43,46,49,53,54,57^ followed by transgender men only (*n*=6),^24,35,42,50,52,58^ or studies that compared transgender women to transgender men (*n*=7).^10,25,26,39,44,45,55^ Six studies compared either transgender men or transgender women to cisgender people.^27,28,38,47,48,56^ Five studies compared transgender people to cisgender people, but did not specify the gender identity of the transgender group, or used suspect and unreliable measures to determine participant's gender identity (see Discussion section).^22,29,34,38,51^

Excluding the seven studies of transgender women of color that were specifically designed to recruit only Black, Latina, or Two-Spirit individuals, and two studies that did not report the racial composition of their samples, studies of transgender women ranged from 47.8% to 95% people of color. Studies of transgender men range from 0.0% to 47.8% people of color.

The results for each inclusion category of our search are reported below.

### Human immunodeficiency virus

Overall, studies reported high rates of HIV among transgender women (range: 10–52.4%). Rates of HIV infection between transgender men and the general population were similar, although some studies reported higher rates (range: 0–11%). Studies also found high rates of condomless receptive intercourse (CRI) among both transgender women and transgender men (Table 1).

**Table 1.** **Human Immunodeficiency Virus**

Citation	Methods	Recruitment, characteristics, & location	Sample size	Primary findings
Benotsch et al.^55^	Survey	Community sample of transgender individuals in Washington, DC, or Richmond.	*n*=112 transgender women	25.2% self-reported HIV+. For transgender women only, using the Internet to meet sexual partners was associated with 11 riskier sexual behaviors, including having multiple partners, sex under the influence of drugs, number of condomless anal or vaginal sex acts, and history of commercial sex work (*p*s<0.05).
			*n*=54 transgender men	
Bradford et al.^10^	Phone survey	Community sample of transgender individuals in Virginia.	*n*=229 transgender women	Eight percent self-reported HIV+.^a^
			*n*=121 transgender men	
Brennan et al.^8^	Structured interview	Community sample of transgender women in Chicago or Los Angeles.	*n*=151 transgender women	Sixteen percent self-reported HIV+. Three or more of the following: low self-esteem, substance use, and intimate partner violence were additively associated with increased incidence of HIV (OR=6.61, *p*<0.05).
Chen et al.^24^	Retrospective chart review	Transgender men from HIV test sites funded by the San Francisco Department of Public Health.	*n*=59 transgender men	None tested positive for HIV. Thirty-nine percent reported CRAI and 51% reported condomless receptive vaginal intercourse in the last 12 months.
Clements-Nolle et al.^25^	Structured interview and HIV test	Community sample of transgender individuals living in San Francisco.	*n*=392 transgender women	Thirty-five percent of transgender women and 2% of transgender men tested positive for HIV. African American race (OR=5.81, *p*<0.001), a history of injection drug use (OR=2.69, *p*<0.001), having more than 200 lifetime sexual partners (OR=2.64, *p*<0.001), and low educational attainment (OR=2.08, *p*<0.01) were associated with a positive HIV test.
			*n*=123 transgender men	
Edwards et al.^27^	Structured interview	Convenience sample of cisgender and transgender individuals utilizing HIV/AIDS programs in Los Angeles.	*n*=107 transgender women	52.44% of transgender participants self-reported HIV+ and were more likely to be HIV+ (OR=3.76, *p*<0.001).
			*n*=2019 cisgender people	
Ellingson and Odo^57^	Structured interview	Convenience sample of Mahuwahine individuals recruited from HIV/AIDS and transgender community organizations in Hawaii.	*n*=128 transgender women	0.07% (1 person) self-reported HIV+.
Flentje et al.^28^	Retrospective chart review	Individuals utilizing substance abuse treatment funded by the San Francisco Department of Public Health.	*n*=146 transgender women	Transgender women were more likely to be tested for HIV than cisgender women (*p*<0.01).
			*n*=4011 cisgender women	
			*n*=53 transgender men	
			*n*=9429 cisgender men	
Fletcher et al.^11^	Survey	Community sample of transgender women using an HIV prevention program in Los Angeles.	*n*=517 transgender women	24.2% self-reported HIV+. HIV+ women more likely to engage in serodiscordant CRAI with casual (AOR=6.7, *p*<0.05) and commercial partners (AOR=16.29, *p*<0.01) compared to the HIV− cohort.
Golub et al.^43^	Survey	Transgender women in New York City Metro area participating in HIV risk reduction intervention.	*n*=75 transgender women	50.7% of the study's cohort reported HIV+ status.
Kellogg et al.^30^	Prospective chart review	Transgender women who had multiple HIV tests at a single site in San Francisco.	*n*=155 transgender women	15.5% seroconverted during the 3-year study. African American race (AHR=5.0, 95% CI=1.5, 16.2) and CRAI (AHR=3.9, 95% CI=1.2, 13.1) were independent predictors of HIV seroconversion.
Kenagy^44^	Structured interview	Transgender individuals recruited from a Philadelphia AIDS community-based organization.	*n*=49 transgender women	18.8% of transgender women and no transgender men self-reported HIV+.
			*n*=32 transgender men	
Kenagy et al.^5^	Structured interview	Community sample of transgender individuals in Chicago or Philadelphia.	*n*=122 transgender women	Transgender men were more likely to have had condomless sex in the last 3 months than transgender women (OR=8.92, *p*<0.001).
			*n*=62 transgender men	
Nemoto et al.^31^	Structured interview	Transgender women of color recruited from HIV organizations in San Francisco.	*n*=332 transgender women	25.9% self-reported HIV+.
Nemoto et al.^32^	Structured interview	Transgender women engaged in sex work in San Francisco or Oakland.	*n*=573 transgender women	29.9% self-reported HIV+.
Nuttbrock et al.^12^	Structured interview and HIV test	Community sample of transgender women in the New York Metro area.	*n*=591 transgender women	40.1% tested positive for HIV. Gender abuse and younger age interacted to increase likelihood of being HIV+ (HR=2.82, *p*<0.05).
Nuttbrock et al.^46^	Prospective structured interview and HIV test	Community sample of transgender women in the New York Metro area determined to be HIV−.	*n*=230 transgender women	2.8% seroconverted during the 3-year study. Gender abuse predicted HIV/STI infection (HR=1.67, *p*<0.05).
Operario et al.^33^	Survey	Community sample of transgender women in a primary partnership with a man who was living in San Francisco.	*n*=174 transgender women	Forty-one percent self-reported HIV+.
Peitzmeier et al.^47^	Retrospective chart review	Individuals who received a pap test at an LGBT health clinic in Boston.	*n*=233 transgender men	0.9% of transgender men and 1.1% of cisgender women were HIV+ (*p*=1.00).
			*n*=3625 cisgender women	
Reisner et al.^49^	Structured interview	Transgender women engaged in sex work in Boston.	*n*=11 transgender women	36.4% self-reported HIV+.
Reisner et al.^50^	Structured interview, qualitative research	Transgender men in Boston who have sex with cisgender men.	*n*=16 transgender men	No one self-reported HIV+ status.
Reisner et al.^52^	Retrospective chart review	Transgender male patients at an LGBT health clinic in Boston.	*n*=23 transgender men	4.3% (1 person) was HIV+.
Reisner et al.^52^	Retrospective chart review	Transgender individuals and a nested, age, race, and SES-matched sample of cisgender individuals accessing care at an LGBT health clinic in Boston.	*n*=31 transgender people	12.9% and 18.6% of the transgender^a^ and matched cisgender cohort, respectively, were HIV+ (*t*=0.50, *p*=0.478).
			*n*=62 matched cisgender women	
			*n*=62 matched cisgender men	
Rowniak et al.^35^	Structured interview	Convenience sample of transgender MSM in San Francisco.	*n*=14 transgender men	7.1% (1 person) self-report HIV+.
Santos et al.^36^	Structured interview and HIV test	Transgender women in San Francisco.	*n*=285 transgender women	Thirty-five percent tested positive for HIV. Methamphetamine and powdered cocaine use before or during anal intercourse was significantly associated with a positive HIV test (AOR=3.27, *p*<0.01 and AOR=3.38, *p*=0.02, respectively).
Santos et al.^36^	Structured interview and HIV test	Community sample of transgender women in San Francisco.	*n*=314 transgender women	39.5% tested positive for HIV. Marginally housed or homeless participants were less likely to be taking ART (AOR=0.03, *p*=0.016) and to be virologically suppressed (AOR=0.05, *p*=0.011).
Sevelius et al.^38^	Structured interview	HIV+ individuals on ART in Milwaukee, Los Angeles, New York City, or San Francisco.	*n*=35 transgender women	Transgender women were less likely to be ART adherent (OR=0.49, *p*=0.05) and also reported less adherence self-efficacy (*t*=2.99, *p*<0.05). Self-efficacy mediated care adherence (*p*<0.001).
			*n*=2770 cisgender men	
Stephens et al.^39^	Retrospective chart review	Individuals accessing care at an STD clinic in San Francisco.	*n*=223 transgender women	Ten percent and 11% of transgender men and transgender women, respectively, were HIV+ (*p*=0.34).
			*n*=69 transgender men	
Sugano et al.^40^	Survey	Transgender women of color engaged in sex work recruited from transgender people of color and AIDS organizations in San Francisco.	*n*=332 transgender women	Twenty-six percent self-reported HIV+.
Taylor et al.^54^	Survey	Community sample of transgender women who have sex with men in New York City who completed a 4-week intervention on stress, stigma, and risk behaviors.	*n*=44 transgender women	Fifty-one percent self-reported HIV+.
Wilson et al.^41^	Survey and HIV test	Community sample of transgender women in San Francisco.	*n*=235 transgender women	44.3% tested positive for HIV.

^a^ Prevalence rates not separated by gender identity (i.e., transgender men vs. transgender women).

AIDS, acquired immune deficiency syndrome; AHR, adjusted hazard ratio; AOR, adjusted odds ratio; ART, antiretroviral therapy; CI, confidence interval; CRAI, condomless receptive anal intercourse; HIV, human immunodeficiency virus; MSM, men who have sex with men; SES, socioeconomic status; STI, sexually transmitted infection.

### Cholesterol and blood pressure screenings

Two studies examined cholesterol or blood pressure (BP). Both were longitudinal studies conducted in California. Neither study found that hormone therapy was associated with clinically significant increases in cholesterol levels or BP (Table 2).^26,34^

**Table 2.** **Cholesterol and Blood Pressure**

Citation	Methods	Recruitment, characteristics, & location	Sample size	Primary findings
Deutsch et al.^26^	Longitudinal study, laboratory findings	Transgender individuals beginning cross-gender hormone therapy at an LGBT health clinic in Los Angeles.	*n*=16 transgender women	For transgender women, estrogen therapy was associated with lower blood pressure (systolic, *p*=0.006 and diastolic *p*=0.001) as well as increased triglyceride (*p*=0.013) and HDL (*p*=0.049), but not LDL levels or weight (*p*s>0.05). Transgender men only had a significant increase in weight (*p*=0.024)
			*n*=31 transgender men	
Roberts et al.^34^	Chart review of laboratory findings	Transgender individuals on cross-gender hormone therapy for more than 6 months accessing care at a clinic in San Francisco.	*n*=55 transgender women	LDL levels in transgender women were lower than those for cisgender men (*p*<0.01) and more closely resembled cisgender women. Triglyceride levels were higher than both cisgender men (*p*<0.01) and cisgender women (*p*<0.005).
			*n*=20 cisgender women	
			*n*=20 cisgender men	

HDL, high-density lipoprotein; LDL, low-density lipoprotein.

### Smoking status

We identified eight relevant articles on tobacco use or smoking status. Most studies reported high rates of tobacco use among transgender people (Table 3). The only study that compared rates of tobacco use among transgender people to a population-based sample of cisgender people found higher rates of smoking among transgender people,^22^ whereas the studies that compared smoking rates among transgender people to cisgender LGB people did not find statistically significant differences in rates of smoking.^29,51,56^

**Table 3.** **Tobacco Use**

Citation	Methods	Recruitment, characteristics, & location	Sample size	Primary findings
Bradford et al.^10^	Phone survey	Community sample of transgender individuals in Virginia.	*n*=229 transgender women	Sixty-four percent reported lifetime tobacco use and 13% reported current use. Reporting a “problem with tobacco” was associated with experiencing discrimination (AOR=1.33, *p*=0.03).^a^
			*n*=121 transgender men	
Conron et al.^22^	Phone survey	Community sample of cisgender and transgender individuals in Massachusetts.	*n*=131 transgender people	Transgender (36.2%) sample more likely to be a current smoker than cisgender (17.3%) sample (*χ*^2^=7.19, *p*<0.01).^a^
			*n*=28,045 cisgender people	
Johnson-Jennings et al.^29^	Survey	Two-spirit American Indian individuals from Seattle, San Francisco, Los Angeles, Denver, Tulsa, Minneapolis, or New York City.	*n*=70 transgender people	45.2% of two-spirit individuals reported daily smoking. Daily smoking was correlated with higher reported stress due to experiences of racial discrimination in the transgender group (*r*=0.12, *p*<0.05).
			*n*=202 cisgender gay/lesbian people	
			*n*=129 bisexual people	
Light et al.^58^	Online survey	Convenience sample of transgender men who had experienced pregnancy after transition.	*n*=41 transgender men and nonbinary people assigned female at birth	7.3% reported smoking during their pregnancy.
Reisner et al.^52^	Retrospective chart review	Transgender male patients at an LGBT health clinic in Boston.	*n*=23 transgender men	Thirteen percent reported current tobacco use.
Reisner et al.^52^	Retrospective chart review	Transgender individuals and a nested, age, race, and SES-matched sample of cisgender individuals accessing care at an LGBT health clinic in Boston.	*n*=31 transgender people	Smoking prevalence did not differ between transgender (54.8%) and matched cisgender (59.7%) participants (*χ*^2^=0.25, *p*=0.620).
			*n*=62 matched cisgender women	
			*n*=62 matched cisgender men	
Sanchez et al.^53^	Structured interview	Transgender women in New York City recruited from HIV/AIDS nonprofit organizations.	*n*=101 transgender women	Fifty percent reported current tobacco use. Participants with a regular healthcare provider were less likely to be current smokers (*p*<0.004).
Tamí‐Maury et al.^56^	Survey	Convenience sample of LGBT individuals recruited at a Houston Pride festival.	*n*=5 transgender people	Four of 5 transgender respondents reported current tobacco use. No statistical test performed due to small transgender sample size.
			*n*=45 cisgender males	
			*n*=44 cisgender females	

^a^ Rates not separated by gender identity (i.e., transgender men vs. transgender women).

### Pelvic health screenings

Four articles addressed Pap tests. Two of these studies assessed self-reported rates of lifetime^42^ or recent screenings^50^ in samples of only transgender men or nonbinary people assigned female at birth. The other two articles compared transgender men to cisgender women, with both finding that transgender men were less likely to be up-to-date on Pap tests (Table 4).^47,48^

**Table 4.** **Pelvic Health**

Citation	Methods	Recruitment, characteristics, & location	Sample size	Primary findings
Dutton et al.^42^	Interview	Convenience sample of transgender men in New Haven, CT.	*n*=6 transgender men or nonbinary people assigned female sex at birth	Five of 6 reported receiving a lifetime pelvic examination.
Peitzmeier et al.^47^	Retrospective chart review	HIV individuals with a cervix who received care at an LGBT health clinic in Boston.	*n*=350 transgender men	Transgender individuals were less likely to be up-to-date on Pap screenings than cisgender individuals (AOR=0.63, *p*<0.01).
			*n*=4882 cisgender women	
Peitzmeier et al.^47^	Retrospective chart review	Individuals who received a pap test at an LGBT health clinic in Boston.	*n*=233 transgender men	Transgender men were more likely to have an inadequate Pap than cisgender women (AOR=10.77, *p*<0.0001). Time on testosterone was associated with higher rates of inadequate Pap for transgender men (AOR=1.19, *p*=0.01). The relationship between transgender identity and inadequate pap remained when controlling for testosterone therapy (AOR=6.01, *p*<0.0001).
			*n*=3625 cisgender women	
Reisner et al.^50^	Structured interview, qualitative research	Transgender men in Boston who have sex with cisgender men.	*n*=16 transgender men	68.8% reported a Pap test in the last 12 months.

### Insurance status

Eight articles assessed insurance status of transgender individuals as secondary research questions part of a larger study. Ranges for insurance coverage varied widely (28.0–90.7% reporting coverage, Table 5).

**Table 5.** **Insurance**

Citation	Methods	Recruitment, characteristics, & location	Sample size	Primary findings
Bazargan and Galvan^23^	Structured interview	Community sample of low-income transgender Latina women in Los Angeles.	*n*=220 transgender women	28.0% reported health insurance coverage.
Bradford et al.^10^	Phone survey	Community sample of transgender individuals in Virginia.	*n*=229 transgender women	70.9% reported health insurance coverage.^a^
			*n*=121 transgender men	
Chen et al.^24^	Retrospective chart review	Transgender men from HIV test sites funded by the San Francisco Department of Public Health.	*n*=59 transgender men	52.5% reported health insurance coverage.
Conron et al.^22^	Phone survey	Community sample of cisgender and transgender individuals in Massachusetts.	*n*=131 transgender people	86.2% of transgender people reported health insurance coverage. 22.8% of transgender people reported public health insurance coverage.^a^ There were no differences in insurance coverage between transgender and cisgender people (all *p*s>0.50).
			*n*=28,045 cisgender people	
Peitzmeier et al.^47^	Retrospective chart review	Individuals who received a pap test at an LGBT health clinic in Boston.	*n*=233 transgender men	90.7% of transgender men had health insurance coverage. Rates of insurance coverage did not differ between cisgender and transgender people (*p*=0.88).
			*n*=3625 cisgender women	
Reisner et al.^50^	Structured interview, qualitative research	Transgender men in Boston who have sex with cisgender men.	*n*=16 transgender men	75.0% reported health insurance coverage.
Sanchez^53^	Structured interview	Transgender women in New York City recruited from HIV/AIDS nonprofit organizations.	*n*=101 transgender women	77.2% reported health insurance coverage. 69.2% of those with insurance had Medicaid.
Santos et al.^36^	Structured interview and HIV test	Community sample of transgender women in San Francisco.	*n*=314 transgender women	Health insurance status was not associated with ART use among HIV+ transgender sample (AOR=1.66, *p*=0.639). Rates of coverage not reported.

^a^ Rates not separated by gender identity (i.e., transgender men vs. transgender women).

## Discussion

### Overall trends

Forty-one unique articles addressed primary care concerns among transgender people. The majority of articles were related to HIV prevalence or risk factors. Careful examination of the sample characteristics reveals several gaps in the literature, discussed below.

### Terminology and language

Four hundred studies were removed from the initial search that erroneously used the keyword “transgender” to describe cisgender LGB people. Describing samples as LGBT inclusive when these samples do not include transgender people reinforces the idea that the health concerns of transgender people are equivalent to those of cisgender LGB people and promotes the incorrect assumption that transgender people cannot also be lesbian, gay, or bisexual. The majority of studies identified transgender people using the now-standard two question approach, which asks “What sex were you assigned at birth OR What was your sex at birth?” and then “What is your current gender?” This two-part question system allows transgender people to correctly identify themselves with minimal confusion.^59^ However, one study that used a phone survey method used “sound of voice” over the phone to determine gender identity, but only for transgender, not cisgender, participants.^22^ Using such a method confounds the validity of the results and underscores the need for consistent reliable methodology in conducting research among transgender individuals. Researchers should take care to accurately describe their sample with regard to sexual orientation and gender identity; this practice begins at study design.

Several studies combined samples of transgender men and transgender women.^10,51^ Although this practice is understandable given limited sample sizes, findings from studies that do not report outcomes or prevalence rates by gender identity are difficult to apply in clinical settings. In general, there is a much smaller literature regarding transgender men. More research should focus on transgender men to better understand their healthcare needs. Furthermore, no study to date has addressed the needs of nonbinary people or the needs of gender nonconforming people. Some studies have included nonbinary people in their samples of transgender men; however, these studies' small sample sizes limit statistical inference.^42,58^ There is a need for studies designed specifically to recruit nonbinary people to assess rates of receipt of primary care, health disparities, and general experiences with healthcare systems.

### Population characteristics

All but two of the studies in this review were conducted in exclusively urban samples. Because health disparities in general are larger for those living in rural areas,^60^ it is likely that transgender people living in rural areas also experience great health disparities. Furthermore, low population density may increase isolation for transgender people and limit access to providers experienced with caring for transgender patients. Only three studies contained samples of transgender people living in the South of the United States. Given the significant regional cultural differences in the United States, particularly regarding religiosity and acceptance of LGBT people,^61^ transgender people living in the South of the United States likely face significant barriers to accessing primary care, as well as high levels of discrimination and isolation.^62^ Studies of transgender people outside of coastal cities are an important opportunity for future research.

Studies of transgender men contained higher percentages of white participants than studies of transgender women. This points to a gap in the literature regarding the experiences of transgender men of color. This gap may be in part explained by gender differences in study design. Many of the studies of transgender men use chart review methods, while more of the studies that used surveys were of transgender women, with recruitment strategies that focused on low-income women of color due to a focus on HIV/AIDS. Thus, much of the literature regarding transgender men emphasizes the experiences of those who accessed healthcare. Transgender men of color may be less likely to access regular primary care than white transgender men, given that cisgender men of color are less likely than white cisgender men to access primary care.^63^ There is a critical need for studies that address the needs of transgender men of color.

### Rates of HIV

Literature on HIV prevalence largely focused on transgender women. Results from the studies that included transgender men suggest that HIV rates are not elevated among transgender men and are much lower than rates among transgender women.

The majority of studies (18 of 31) used self-report to determine HIV status. Self-report of HIV status likely results in underestimates of HIV prevalence; still, most studies reported extremely high rates of HIV prevalence among transgender women, with reported rates varying from 16% to more than 50%. Most of these studies were not designed to address true population-based prevalence rates because they used convenience sampling methods or focused on specific subpopulations (i.e., sex workers). There is a dearth of national data on rates of HIV in transgender people, especially transgender women, as most research is limited to large urban areas. One article on the global burden of HIV on transgender women estimates that 21.7% of transgender women in the United States are HIV positive and most of this risk is associated with condomless sex with cisgender men.^17^ A number of recent reviews have addressed the prevalence of HIV specifically in transgender women, finding that, worldwide, transgender women engaged in sex work are at significantly higher risk for contracting HIV than cisgender people engaged in sex work.^20^ There is an estimated worldwide HIV prevalence rate of 19.1% among transgender women.^17^ Based on the findings from this systematic review and other reviews that included international samples, there are specific populations of transgender women who are at greater risk for contracting HIV.^19^ Being African American is independently associated with increased likelihood of being HIV positive,^25,30^ as is engaging in sex work,^55^ illicit drug use,^25,36,55^ and experiencing gender-based violence.^12,46^ Interestingly, one study used a cisgender comparison sample matched for age, race, and socioeconomic status (SES) and found no significant differences in rates of HIV between groups,^51^ highlighting the importance of considering comparison samples when conducting transgender health research. In sum, growing evidence suggests that there is an HIV/AIDS syndemic among transgender women, such that structural factors and marginalization lead to increased incidence of HIV.^8^

### Sexual behavior and HIV

Both transgender men and women have high rates of sexual practices that increase risk for HIV infection. A study in 2005 found only 29% of transgender male respondents, and 59% of transgender female respondents used protection during their last sexual encounter.^45^ Reported rates of condomless receptive anal intercourse (CRAI) vary widely by study, in part due to differences in sample demographics but also in the manner in which CRAI self-reports are assessed. For example, one study reported a 90.9% lifetime incidence of CRAI,^49^ and two studies found that more than 75% of their transgender sample reported condomless receptive sex in the last 3 months.^35,44^ However, most studies that assess for CRAI in the last 2–3 months report ranges from 30% to 50% among transgender people.^24,33,50,57^

Another study reported higher rates of condomless intercourse among transgender men than women,^45^ although transgender men overall do not show high rates of HIV infection. This may be due to differences in HIV transmission risk between receptive anal and receptive vaginal intercourse, wherein CRAI is associated with an estimated 10–20 times greater risk of HIV contraction than condomless vaginal intercourse,^64–66^ differences between groups in types of sexual partners (commercial sex work, casual sexual partners, or primary partners), or differences in healthcare utilization, as there is some evidence for higher rates of healthcare utilization among transgender men than transgender women.^14^ Future studies should assess differences between the sexual practices and attitudes of transgender men and women to determine why both groups show such high rates of CRI, but only transgender women show high rates of HIV infection.

Several studies also addressed risk factors related to increased likelihood of engaging in CRAI. Factors associated with CRAI include living with a primary sexual partner, having a sexual partner who is an injection drug user, and sexual partner seroconcordance^33,41^ as well as intimate partner violence and substance use.^8,31^ Being HIV positive was also associated with increased likelihood of CRAI, independent of partner status,^11,31^ as was the use of Internet dating websites or apps.^55^ Those with high self-efficacy or self-esteem are less likely to engage in CRAI.^32,40,43^ Engaging in CRAI may be a survival tactic for individuals who live at the intersection of multiple marginalized identities marked by social instability and limited access to resources.^32^ In addition to the substantial and growing quantitative literature regarding transgender people and HIV risk behaviors, more qualitative work is needed to better understand the experiences and needs of transgender women engaging in HIV risk behaviors.

### HIV screening practices

The CDC recommends that all people at high risk for HIV infection undergo HIV screening at least annually.^67^ Transgender people undergo HIV screenings at higher rates than the general population. In an analysis of the Massachusetts' 2007–2009 BRFSS, 16.8% of the transgender survey respondents reported having an HIV test during the last year, compared to only 9.8% of cisgender respondents who reported having an HIV test during the last year.^22^ In a 2010 study focused on lifetime HIV testing in transgender men living in Boston, 94% of study participants reported having an HIV test at some point in their life.^50^ Another study in San Francisco found transgender women were more likely to be tested for HIV than cisgender women,^28^ and a study of individuals involved in the Los Angeles House Ball scene found 83% of participants had been tested for HIV in the last 6 months, although this study did not distinguish transgender people from cisgender people statistically.^68^ Screening for HIV—and other STDs—remains an important tool in attending to the primary care needs of transgender people. This remains true even for HIV-positive individuals. In a large cohort study of transgender women in New York City, there was a substantial risk of incident sexually transmitted infection (STI) following HIV diagnosis, underscoring the importance of frequent STI screening and prevention counseling for individuals who engage in high-risk behavior.^69^

### Barriers to HIV care and HIV interventions

There has been little research addressing transgender-specific barriers to HIV prevention; much of the current HIV literature among transgender women focuses on behavioral correlates of either CRAI or HIV-positive status. However, a recent qualitative study of a national sample of transgender women identified several barriers to accessing HIV preventive care. These barriers include stigma, being unaware or misinformed about resources, exclusion from relevant clinical research trials, mistrust of the scientific community, and perceived possible HIV-vaccine side effects. Facilitators to accessing necessary preventive resources included increased information and awareness, research staff and providers sensitive to transgender individual's needs, recommendations from a trusted transgender-friendly healthcare provider, and assistance with basic needs.^70^

There is a small body of research on the effectiveness of various HIV prevention program interventions among transgender populations.^43,54,71^ Recommendations for future HIV harm reduction programs include targeted advertising on Internet hook up sites and attention to the housing status of participants.^11,55^ Housing status is particularly important because research demonstrates an unstable housing environment is associated with lower treatment adherence in HIV-positive transgender women.^11,37,38^ A final recommendation is made by Bockting et al. who argue that facilitating interaction between transgender peers in HIV prevention initiatives, in addition to promoting safer sex behaviors, deflects the impact of gender-based abuse and violence.^72^ Given the link between gender-based violence and increased incidence of HIV as well as HIV risk behaviors, reducing gender-based violence is essential to HIV harm reduction initiatives.^12,46^

### Cholesterol screening

The American Heart Association recommends that all adults, beginning at age 20, undergo cholesterol screening every 2 years, with those at a higher risk for heart disease screened more frequently.^73^ In the present review, two studies addressed cholesterol levels and screenings, with both being longitudinal studies investigating gender affirming hormone therapy on transgender people's body mass index (BMI), BP, and lipids. A 2014 chart review study examined cholesterol levels for transgender women following hormone therapy and found that low-density lipoprotein levels resembled cisgender female values, but triglyceride levels were higher than cisgender males and females. High-density lipoprotein levels were not statistically different between transgender women and either cisgender group. The authors conclude that current clinical laboratory reference guidelines for transgender women may not be clinically helpful, as transgender patients in their study did not fall within the established guidelines for cisgender women on several measures, and because these guidelines are based on healthy limits for postmenopausal women receiving estrogen hormone therapy.^34^ Meanwhile, Deutsch and colleague's 2015 study suggests that transgender people receiving gender-affirming hormone therapy do not develop abnormal BP or lipids levels in the short term. In this study, gender-affirming hormone therapy was associated with a reduction in BP in transgender women and increased BMI in transgender men.^26^ Taken together, these two studies underscore the importance of hormone level monitoring to guide therapy. More research is needed to address the potential need for clinical reference guidelines relevant to transgender people undergoing gender-affirming hormone therapy.

### Tobacco use

There is a paucity of research on tobacco use in transgender communities, but rates are likely higher than the general population.^28^ A 2009 study of transgender women living in New York City found 50% of interviewed participants reported current tobacco use.^53^ Furthermore, in an analysis of Massachusetts' 2007–2009 BRFSS data, transgender people were more likely to report that they were a current smoker (26%) than cisgender people (17%).^22^ In a large study of transgender people living in Virginia, rates of tobacco use were also high. Of the study's 350 eligible survey respondents, 64% reported tobacco use at some point in their life, 23% reported having a lifetime problem with tobacco, and 13% reported a current problem with tobacco use.^10^ In a 2015 study of 241 transgender women in San Francisco, 83% indicated having smoked a cigarette in the last month. Of these women, 62.3% reported daily smoking and 51.7% reported an unsuccessful quit attempt. The data also demonstrated that healthcare, employment, and housing discrimination were positively associated with current smoking status, unsuccessful attempts at smoking cessation, and never having attempted cessation of smoking habits.^74^

These above findings differ from an earlier 2001–2002 study of transgender people in Boston, which demonstrated no difference in smoking status between transgender and cisgender study populations.^51^ This difference illustrates the importance of comparison populations in transgender healthcare literature. Specifically, differences in findings between studies are likely due to sample characteristics; the Reisner et al. study used an age, race, and SES-matched sample of cisgender individuals seeking care at the same LGBT clinic, whereas other studies, including the BRFSS Massachusetts survey study, compared transgender people to a large mixed sample of heterosexual people. Given the high rates of tobacco use among cisgender LGB people,^75^ the differences between studies are interesting and suggest that tobacco use rates among transgender people may be higher than the general population, but not higher than rates among cisgender LGB people.

Finally, most studies did not address smoking cessation or provider screenings for smoking status. One study did find that transgender women with a regular healthcare provider were less likely to be current smokers than those without a provider, although this could simply mean that this subset of transgender women has more resources to invest in their long-term health or are more health conscious.^56^ More studies are needed to determine the efficacy of smoking cessation interventions for transgender people.

### Pap tests

A growing body of literature suggests lower rates of Pap tests among transgender men. The U.S. Preventive Services Task Force 2012 guidelines for cervical cancer screening recommend that cisgender women be screened every 3 years starting at age 21 and that women ages 30–65 be screened every 5 years, in combination with HPV testing.^76^ A study of New England FTM transgender men who have sex with cisgender men found 31% of its respondents had not had a Pap test in the last 12 months.^50^ Chart reviews of transgender men living in New England found they were significantly less likely to be up-to-date on Pap tests compared to cisgender female patients.^47,48^ In addition, recent evidence suggests increased rates of insufficient or unsatisfactory samples for Pap tests in transgender men compared to cisgender women, which could be related to cervical tissue changes following androgen exposure, as well as provider discomfort with performing the examination on transgender men.^48^ The low rate Pap tests among transgender men and assigned-female nonbinary individuals are likely due to discomfort surrounding pelvic anatomy, high rates of trauma compounding gender dysphoria-based discomfort, and lack of access to providers with experience seeing transgender patients.^42,48,77^ Coupled with the high rates of condomless receptive sex among transgender men who have sex with cisgender men,^35,44,45^ transgender men may face significant risks for cervical cancer that remains undetected due to decreased screening. There is an urgent need for interventions that focus on the sexual health needs of transgender men, as well as changes in clinic policies that allow transgender men to feel empowered to access sexual healthcare screenings.

### Insurance status

This literature review did not identify any large-scale studies on insurance coverage among transgender people, and we therefore cannot infer overall rates of insurance coverage. The most recent National Transgender Discrimination Survey reports that 19% of transgender people have no health insurance, which is higher than the general U.S. population. Insurance coverage had a significant impact on primary care access; transgender people without insurance are much more likely to report delaying primary care than those with insurance (88% vs. 39%).^9^ In the present literature review, eight studies included observational data about rates of health insurance coverage.^10,22–24,37,48,50,53^ These studies indicate that a large number of transgender people remain uninsured. For transgender people who do have insurance, they are more likely than cisgender people to be covered through public options.^9,53^ Given geographic differences in Medicaid expansion, regional differences in health insurance coverage may be more pronounced among transgender people.

### Primary care gaps in the literature

There were no studies that addressed breast or chest tissue screenings, rates of receipt of annual flu shots, or colorectal screenings among transgender people. With regard to breast/chest tissue health, a recent US retrospective study of 5135 transgender people receiving gender-affirming hormone therapy found that hormone treatment in both male- and female-assigned people did not result in a greater incidence of breast cancer compared to the general population.^78^ A qualitative study of six transgender men found that half of participants reported significant discomfort regarding their chest tissue and that half reported “binding” their chest tissue on a regular basis.^42^ However, there is no research literature addressing how discomfort with anatomy may impact access to screenings for transgender people. Transgender women should receive chest tissue screenings following a minimum of 5 years of hormone therapy, regardless of age, and transgender people who undergo bilateral mastectomy, chest reduction, or “top” surgery and still have remaining chest tissue should undergo cancer screenings as recommended for cisgender women.^79,80^ Flu shots are an important component of primary healthcare and are of particular importance among populations with high incidence of HIV/AIDS.^81^ There is no research literature that addresses receipt of flu shots among transgender people, although receipt of flu shots could serve as a simple metric of access to care in future studies. Finally, the lack of studies examining colorectal care highlights another significant gap in the transgender health literature: the inclusion of transgender elders. Most studies in the present review report mean ages between 30 and 40. The health needs of transgender elders are largely unaddressed by the research literature and represent an important area of opportunity for inquiry and intervention.

## Conclusion

By performing a systematic literature review, we identified several opportunities for future research that could improve care for transgender individuals, particularly regarding representation of rural and Southern populations, transgender men of color, and transgender elders. With increasing visibility of transgender people comes a growing ethical imperative for clinicians and researchers to meet the needs of this diverse population. The findings of the present literature review indicate that preventive healthcare among transgender populations is less emphasized than other research areas; even less research has focused on developing health promotion or healthcare utilization interventions to reduce health disparities among transgender people. As the transgender healthcare literature comes of age, more research should focus on developing interventions to serve transgender people, while also considering approaches that include transgender people using participatory designs to ensure that the needs of transgender communities are well represented in the research literature.^82^

## Abbreviations Used

AIDS: acquired immune deficiency syndrome
BMI: body mass index
BP: blood pressure
BRFSS: Behavioral Risk Factor Surveillance System
CRAI: condomless receptive anal intercourse
CRI: condomless receptive intercourse
HIV: human immunodeficiency virus
HPV: human papillomavirus
LDL: low-density lipoprotein
SES: socioeconomic status
